# Regulation of Hair Follicle Growth and Development by Different Alternative Spliceosomes of FGF5 in Rabbits

**DOI:** 10.3390/genes15040409

**Published:** 2024-03-26

**Authors:** Shaoning Sun, Bohao Zhao, Jiali Li, Xiyu Zhang, Shuyu Yao, Zhiyuan Bao, Jiawei Cai, Jie Yang, Yang Chen, Xinsheng Wu

**Affiliations:** 1 College of Animal Science and Technology, Yangzhou University, Yangzhou 225009, China; 19952323987@163.com (S.S.); bhzhao@yzu.edu.cn (B.Z.); li1193117036@163.com (J.L.); zxy478002497@163.com (X.Z.); yaoshuyu321@163.com (S.Y.); 18352764997@163.com (Z.B.); cjwcjw520800@163.com (J.C.); 19851563850@163.com (J.Y.); yangc@yzu.edu.cn (Y.C.); 2 Joint International Research Laboratory of Agriculture & Agri-Product Safety, Yangzhou University, Yangzhou 225009, China

**Keywords:** angora rabbit, FGF5 gene, alternative spliceosome, hair follicle growth

## Abstract

This study investigated the regulatory effect of alternative spliceosomes of the fibroblast growth factor 5 (*FGF5*) gene on hair follicle (HF) growth and development in rabbits. The FGF5 alternative spliceosomes (called *FGF5-X1*, *FGF5-X2*, *FGF5-X3*) were cloned. The overexpression vector and siRNA of spliceosomes were transfected into dermal papilla cells (DPCs) to analyze the regulatory effect on DPCs. The results revealed that *FGF5-X2* and *FGF5-X3* overexpression significantly decreased *LEF1* mRNA expression (*p* < 0.01). *FGF5-X1* overexpression significantly reduced *CCND1* expression (*p* < 0.01). *FGF5-X1* and *FGF5-X2* possibly downregulated the expression level of *FGF2* mRNA (*p* < 0.05), and FGF5-X3 significantly downregulated the expression level of *FGF2* mRNA (*p* < 0.01). The FGF5 alternative spliceosomes significantly downregulated the *BCL2* mRNA expression level in both cases (*p* < 0.01). *FGF5-X1* and *FGF5-X2* significantly increased *TGFβ* mRNA expression (*p* < 0.01). All three FGF5 alternative spliceosomes inhibited DPC proliferation. In conclusion, the expression profile of HF growth and development-related genes can be regulated by FGF5 alternative spliceosomes, inhibiting the proliferation of DPCs and has an influence on the regulation of HF growth in rabbits. This study provides insights to further investigate the mechanism of HF development in rabbits via *FGF5* regulation.

## 1. Introduction

Wool production is a fundamental trait of the rabbit industry in terms of economy. The hair follicle (HF), identified as a complex and vital organ that determines the cyclic process in mammals, is the key factor that determines hair growth and development in mice [1], rabbits [2], and sheep [3]. Rabbit hair is a type of natural protein fiber and is one of the high-grade textile raw materials used in the light textile industry [4]. To promote the development of the rabbit hair industry, the regulation and growth conditions of hair follicles and related growth factors must be explored.

The hair follicle cycle is a developmental process that outlives other organs during the whole life of mammals. Hair follicles need to undergo a complex process regulated by multiple signaling pathways. They need to pass through anagen, catagen, and telogen phases, until the end of follicle life [5,6]. During the growing period, a dynamic transition period, DPCs form hair shafts, the hair follicles deepen, and the hair nipples grow to the dermis, thereby providing nutrients for the growing hair. Based on the signal stimulation received, hair follicle growth ends and enters the retrogression period. During the retrogression period, the hair follicle regenerates itself, the blood supply to the hair is interrupted, the hair nipple gradually shrinks, the hair stops growing, and the hair length increases; mainly, the hair follicle depth decreases. At the same time, melanin production in the hair follicles gradually decreases, and hair follicle melanocytes develop apoptosis [7]. The hair follicle then enters the telogen phase, and the epidermal cells attached to the hair follicle maintain normal growth and accumulate at the base of the hair follicle. This allows the hair to maintain its natural state, even in the absence of nutrients provided by the growth phase, until the hair follicles regrow. The hair root from the hair follicle base, along with hair shedding. Finally, the hair shaft reappears at the end of the telogen phase, and the follicle enters a new follicle cycle. Hair follicles are evenly distributed on the skin during embryonic development, thus exhibiting a regular arrangement. Hair follicles are formed because of the interaction of the epidermis and mesenchymal cells and run through the epidermis and dermis. Hair follicles are of two types: primary and secondary. Primary hair follicles occur earlier and have a more complex structure than secondary hair follicles. Hair follicles are distributed in groups, with primary hair follicles arranged neatly on one side and secondary hair follicles surrounding the distribution of primary hair follicles.

In alternative splicing, an identical mRNA precursor is spliced into different mRNA fragments. These fragments are transcribed and translated to produce proteins with different functions. Alternative splicing is a crucial molecular mechanism that regulates the protein and genetic diversity of eukaryotes, controlling their developmental process in different tissues, developmental stages, and environments [8]. Different transcripts are generated through alternative splicing, and different alternative spliceosomes may encode different proteins. This increases the richness of the proteome. Alternative spliceosomes offer a high degree of evolutionary plasticity [9,10], and aberrant splicing of splicing factors can serve to regulate human cancer-specific splicing patterns [11]. Meanwhile, alternative spliceosomes may also be used in infertility treatment [12]. The alternative spliceosome is one of the molecular mechanisms of heat stress response in mammals. It offers theoretical support for the study of heat tolerance in mammals [11]. Previous studies have demonstrated that alternative spliceosomes have diverse effects on protein expression and affect the physiological state of various organisms.

In the 1930s, it was discovered that tissue extracts from the brain and pituitary gland contained an active substance that promotes fibroblast growth, but this substance was not isolated and purified until the 1970s. Given the substance’s role, it is called the fibroblast growth factor *(FGF*) [13]. *FGF* is a large family of 23 closely related genes in mammals [14,15]. FGFs are polypeptides of 150–200 amino acids, with 20–50% of the amino acid sequences being identical. Approximately 120 amino acid sequences form highly homologous central regions (50–70%). *FGF1* expression was significantly associated with lymphatic invasion, metastasis, and differentiation [16]. The *FGF2* collagen matrix enhances mitotic activity for skeletal muscle regeneration [17]. *FGF2* and *FGF9* can both alleviate liver diseases clinically [18,19], while FGF8 and FGF15 have opposing roles in mouse forebrain development [20]. For many years, *FGF2* and *FGF7* have been used as clinical drugs for skin ulcer treatment [21]. A study suggested that the mRNA product of the *FGF5* gene is involved in the HF cycle [22]. *FGF5*, an FGF family member, serves as the hair growth inhibitor [23]. *FGF5* acts as an inhibitor for regulating the transition of DPCs from the anagen to the degenerative phase in the cashmere goat, and *FGF5* knockdown leads to longer wool [24]. The results of research on the *FGF* family are valuable. However, there are currently few reports on the regulation of hair follicle development by FGF5 alternative spliceosomes.

At present, the molecular function of *FGF5* in mice and humans has been reported, but there is not much evidence on the FGF5 alternative spliceosome in rabbits [25]. In this study, the alternative spliceosomes of *FGF5-X1*, *FGF-X2*, and *FGF-X3* were cloned, and the biological functions of these alternative spliceosomes were investigated. The spliceosomes could regulate DPC cell proliferation. Overexpression and knockdown of these spliceosomes could disturb the expression level of HF growth- and development-related genes. This study aims to explore the regulatory effect of *FGF5* gene alternative spliceosomes on rabbit hair follicles and provide a basis for related research on FGF5 alternative spliceosomes. The study provides theoretical aspects for the improvement of rabbit wool production and the treatment of human hair diseases.

## 2. Materials and Methods

### 2.1. Animals

Six-month-old Wanxi Angora rabbits were selected for this experiment. All rabbits were provided by the Rabbit Breeding Base of Anhui Academy of Agricultural Sciences. We randomly selected three rabbits of similar weight (mean weight 2.8 kg) from 120 populations. An approximately 1 cm^2^ area of the rabbit dorsal skin tissue was removed by using autoclaved surgical scissors. The tissue was stored in cryopreservation tubes, labeled, and placed in liquid nitrogen for subsequent DNA and RNA extraction. All the experimental protocols were conducted as per the guidelines provided by the Animal Care and Use Committee at Yangzhou University.

### 2.2. Cell Culture and Transfection

DPCs were grown in mesenchymal stem cell medium (MSCM) (Gibco^®^, Carlsbad, CA, USA) and incubated at 37 °C under 5% CO_2_. The Rab-9 cell line was purchased from American Type Culture Collection (ATCC). In a 24-well plate, cells were uniformly spread and cultured overnight. On reaching 60% confluence, cells were transfected using Lipofectamine™ 3000 (Vazyme, Nanjing, China), according to the manufacturer’s instructions.

### 2.3. FGF5 Alternative Spliceosome Cloning and Plasmid Vector Construction

Total RNA from the skin of the rabbits was extracted as per the directions of the RNAsimple Total RNA Kit (Tiangen, Beijing, China). According to the mRNA sequence of the FGF5 alternative spliceosomes, the primers for the CDS sequences of these spliceosomes were designed using Primer Premier5.0 (Table 1). To clone the CDS sequence of the FGF5 variable spliceosome, the first strand cDNA synthesis kit PrimeScriptTM (Takara, Ichikawa, Japan) was used to synthesize rabbit skin cDNA. The CDS-amplified region of the spliceosomes and pcDNA3.1(+) were ligated through double digestion with *NheI* and *XbaI* and then transformed into receptor cells following recombination. The plasmid was purified and sent to sequencing for identification. The siRNA primers were designed and obtained from Suzhou GenePharma Co., Ltd. (Suzhou, China). Primer sequences are as follows:

Forward Primer: 5′-GCAUCGGUUUCCAUCUGCATT-3′;
Reverse Primer: 5′-UGCAGAGUUAAACCGAUGCTT-3′;
siRNA-NC Forward Primer: 5′-UUCUCCGAACGUGUCACGUTT-3′;
siRNA-NC Reverse primer: 5′-ACGUGACACGUUCGGAGAATT-3′.

### 2.4. Bioinformatics Analysis of FGF5 Alternative Spliceosomes

MegAlign (V17.3.1) and SeqMan (V17.3.1) software in the DNAStar package was used for analyzing the coding sequence of the FGF5 alternative spliceosome. The bioinformatics analysis of the spliceosomes was performed using online websites. ProtParam (https://web.expasy.org/protparam/, accessed on 31 March 2023) was used to predict protein isoelectric points, molecular formula, molecular weight, and instability factor [26]. The hydrophilicity and hydrophobicity of the amino acid sequences were predicted by ProtScale (https://web.expasy.org/protscale/, accessed on 31 March 2023) [27]. The protein secondary structure was analyzed by Prabi (https://npsa-prabi.ibcp.fr/cgi-bin/npsa_automat.pl?page=/NPSA/npsa_server.html, accessed on 17 April 2023) [28]. The protein tertiary structure was constructed using SWISS-MODEL (http://www.swissmodel.expasy.org/, accessed on 17 April 2023) [29]. NCBI (hhttps://www.ncbi.nlm.nih.gov/cdd/, accessed on 10 April 2023) was used to predict the protein structural domain. TMHMM (https://services.healthtech.dtu.dk/services/TMHMM-2.0/, accessed on 1 April 2023) was used for analyzing the protein transmembrane region [30]. Signal peptide prediction was analyzed using Sig-nalP6.0 (https://services.healthtech.dtu.dk/services/SignalP-6.0/, accessed on 17 April 2023) [31]. The phosphorylation site was analyzed using the online tool NetPhos3.1Server (https://services.healthtech.dtu.dk/services/NetPhos-3.1/, accessed on 17 April 2023) [32].

### 2.5. Quantitative RT-PCR

The DPCs were collected using the HiScript^®^III RT SuperMix for qPCR (+gDNA wiper) (Vazyme). Quantitative real-time PCR was performed using the ChamQ SYBR qPCR Master Mix (Vazyme), as per the instructions provided by the manufacturer. Result analyses were performed using QuantStudio^®^ 5 (Applied Biosystems, ABI, Waltham, MA, USA). Primer Premier 5.0 software was used for designing gene primers (Table 2), and *GAPDH* was used as the reference control. The data were organized using Excel 2020, and the relative gene expression was determined using the 2^−ΔΔCt^ method.

### 2.6. Cell Proliferation Assay

CCK-8 Cell Counting Kit (Vazyme) was used to determine the cell apoptosis rate as per the instructions provided by the manufacturer. FACS was performed using the flow cytometer (Becton Dickinson, Macquarie Park, Australian). Cells in good condition were selected and spread on 96-well plates to detect cell proliferation at 0, 24, 48, and 72 h. Then, 10 μL of the CCK-8 reagent was added to each well, and the OD_450_ values were determined. Finally, the cell proliferation curve was plotted.

### 2.7. Statistical Analysis

Statistical analyses were performed via SPSS26.0 (SPSS, Chicago, IL, USA) software. The relative expression of genes was analyzed by paired sample *t*-test. *P* < 0.05 was considered as significant. The results show that all the error lines represent the mean ± standard deviation, and at least three biological replicates were maintained for each group of tests (*n* = 3). The data in this article are the experimental data that have been processed.

## 3. Results and Analysis

### 3.1. Bioinformatics Analysis of Alternative Spliceosomes of FGF5

**Figure 1:**
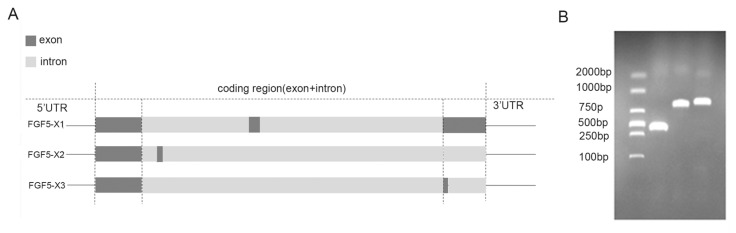
(**A**) A schematic representation of exons of FGF5 alternative spliceosomes. (**B**) FGF5 alternative spliceosome sequence analysis.

**Figure 2:**
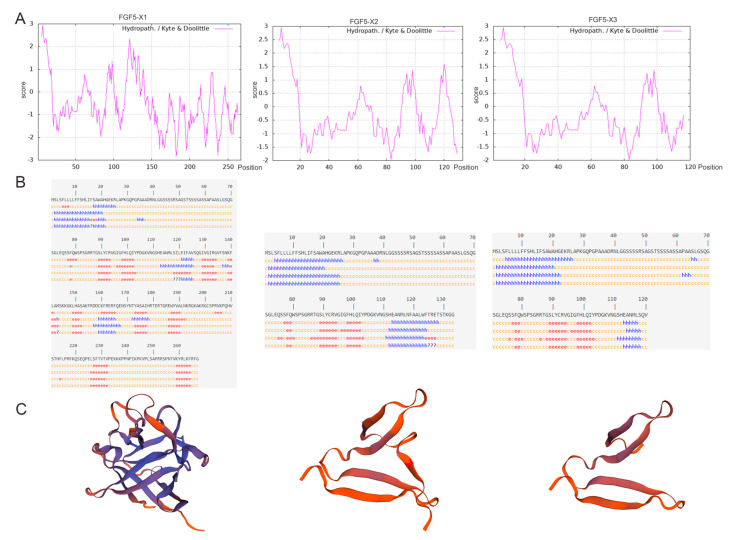

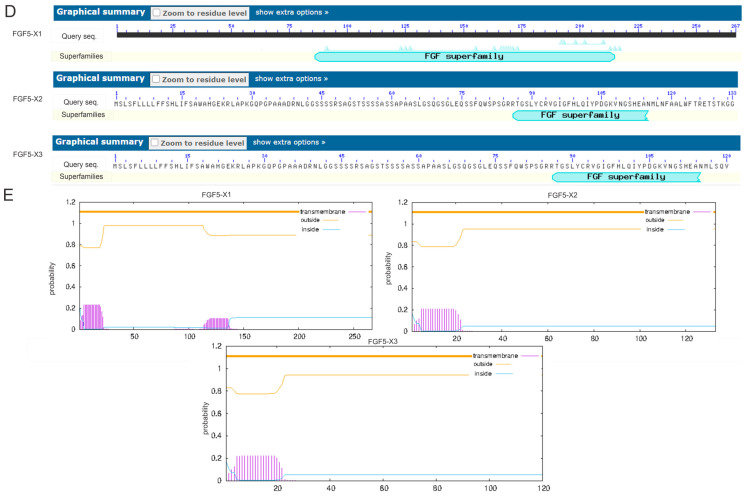

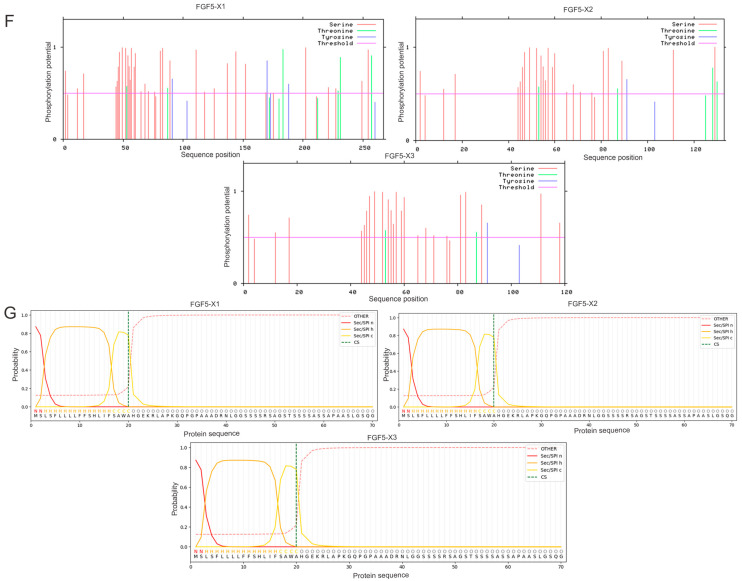
Bioinformatics analysis of FGF5 alternative spliceosomes. (**A**) The hydrophobic folding diagram of the FGF5 alternative spliceosome protein, with the amino acid sequence in horizontal coordinates and the hydrophilic and hydrophobic coefficients in vertical coordinates. (**B**) Prediction of the secondary structure of protein; h, e, and c denote α-helix, strand, and random coil, respectively. (**C**) Prediction of the tertiary structure of FGF5 alternative spliceosome protein. (**D**) Protein structural domain prediction. (**E**) Prediction of the transmembrane domain of FGF5 alternative spliceosome protein. (**F**) Protein phosphorylation site prediction map. (**G**) Prediction of the transmembrane domain of FGF5 alternative spliceosome protein.

The FGF5 alternative spliceosomes were cloned. Of them, *FGF5-X1* contains three exons, and both *FGF5-X2* and *FGF5-X3* contain two exons (Figure 1A). In this study, the FGF5 alternative spliceosomes, the CDS sequences of *FGF5-X1*, *FGF5-X2*, and *FGF5-X3* were first cloned, obtaining 804, 402, and 363 bp sequences, respectively (Figure 1B). The number of amino acids, theoretical isoelectric point, atomic number, and instability coefficient of the proteins encoded by the three FGF5 alternative spliceosomes were predicted using ProtParam online software (1.1, 5/4/7) (Table 3). The instability index estimated FGF5 alternative spliceosomes as an unstable protein of an index of 48.21, 50.84, and 55.20, respectively. The strong hydrophobicity site of *FGF5-X1* was located at 7, with a value of 2.956, amino acid 182 site, and strong hydrophilicity (−2.833). The maximum hydrophilicity of *FGF5-X2* was 2.956, and its minimum value was −1.967. The strongest hydrophobicity value of *FGF5-X3* was 2.956, and the strongest hydrophilicity value was −1.967 (Figure 2A).

The secondary structures of the FGF5 alternative spliceosome proteins were predicted, with results revealing that, in *FGF5-X1*, 267 amino acids contained 26.59% α-helix, 16.48% stretch chain, β-turn 7.12%, and 49.81% random curl. In *FGF5-X2*, 78 (58.65%), 35 (26.32%), 2 (1.50%), and 18 (13.53%) amino acids formed a random coil, an α-helix, β-turn, and an extended strand, respectively. In *FGF5-X3*, 69 (57.50%), 32 (26.67%), 17 (14.17%) and 14 (11.66%) amino acids formed a random coil, an α-helix, β-turn, and an extended strand, respectively (Figure 2B). Tertiary structures of the FGF5 alternative spliceosomes were predicted using SWISS-MODEL software. The results demonstrated that the model maps of different alternative spliceosomes all differed considerably (Figure 2C).

As predicted by the NCBI website, all alternative spliceosomes have no structural domains and belong to the large FGF family (Figure 2D). As predicted by the TMHMM server, the FGF5 alternative spliceosomes contained no transmembrane domain (Figure 2E).

The NetPhos3.1 server predicted that 9 threonine, 34 serine, and 6 tyrosine amino acids of the FGF5-X1 protein had putative phosphorylation sites. Meanwhile, the FGF5-X2 protein was predicted to store 31 phosphorylation sites, including 24 serine, 5 threonine, and 2 tyrosine. Similarly, *FGF5-X3* was predicted to contain 28 phosphorylation sites, with serine 24, threonine 2, and tyrosine 2. However, the FGF5 alternative spliceosome proteins had putative phosphorylation sites (Figure 2F). The probability of signal peptides for *FGF-X1*, *FGF-X2*, and *FGF-X3*, respectively, was 0.8634, 0.8634, and 0.8634 (Figure 2G).

### 3.2. Regulation of HF Growth- and Development-Related Genes by FGF5 Alternative Spliceosomes

**Figure 3:**
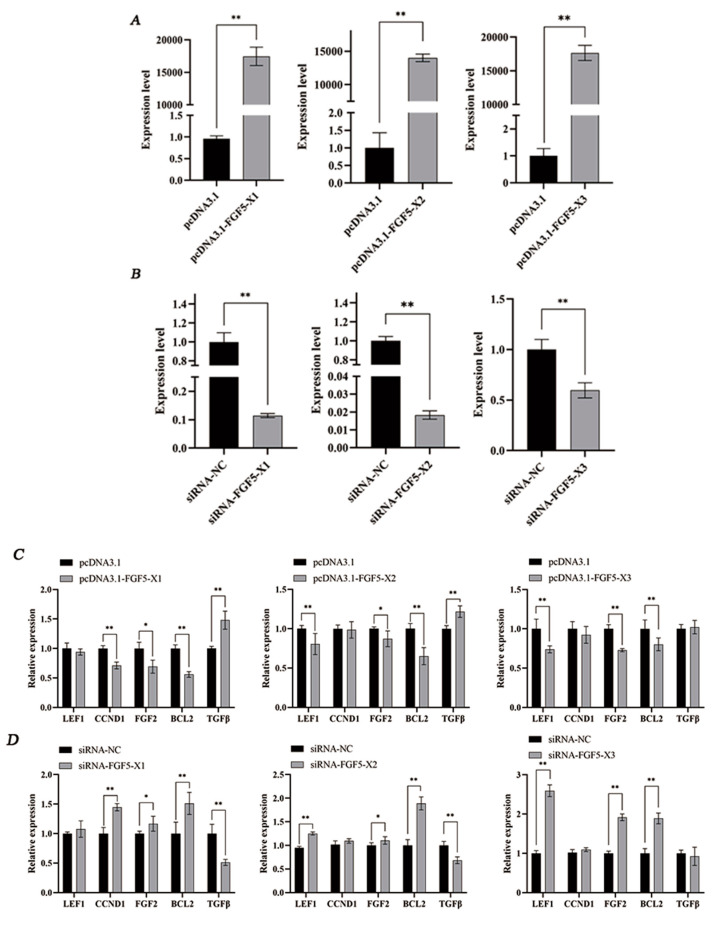
(**A**) pcDNA3.1 of the FGF5 alternative spliceosomes significantly increased the mRNA expression levels in DPCs. (**B**) siRNA of the FGF5 alternative spliceosomes significantly decreased the mRNA expression levels in DPCs. Overexpression (**C**) and knockdown (**D**) of the FGF5 alternative spliceosomes regulate the mRNA expression of skin-related genes. * *p* < 0.05 and ** *p* < 0.01.

According to the results, pcDNA3.1-FGF5-X1, pcDNA3.1-FGF5-X2, and pcDNA3.1-FGF5-X3 overexpression significantly increased FGF5 alternative spliceosome expression (*p* < 0.01, Figure 3A), and *FGF5-X1*, *FGF5-X2*, and *FGF5-X3* gene expression was highly significantly downregulated by siRNA-FGF5-X1, FGF5-X2, and FGF5-X3 in DPCs (*p* < 0.01, Figure 3B).

Moreover, mRNA expression of the HF growth- and development-related genes was detected after the FGF5 alternative spliceosomes were overexpressed and knocked down in DPCs (Figure 3C,D). The overexpressed FGF5 alternative spliceosomes were treated with DPCs. *FGF5-X1* highly significantly reduced *CCND1* expression (*p* < 0.01). *FGF5-X2* and *FGF5-X3* highly markedly downregulated *LEF1* mRNA expression (*p* < 0.01). Furthermore, *FGF1-X1* and *FGF5-X2* significantly downregulated the *FGF2* mRNA level (*p* < 0.05). *FGF5-X3* highly significantly downregulated the *FGF2* mRNA level (*p* < 0.01). In addition, FGF5 alternative spliceosomes may downregulate the *BCL2* mRNA expression level (*p* < 0.01). *FGF5-X1* and *FGF5-X2* significantly increased the *TGFβ* mRNA expression level (*p* < 0.01).

### 3.3. FGF5 Alternative Spliceosomes Inhibit DPC Proliferation

**Figure 4:**
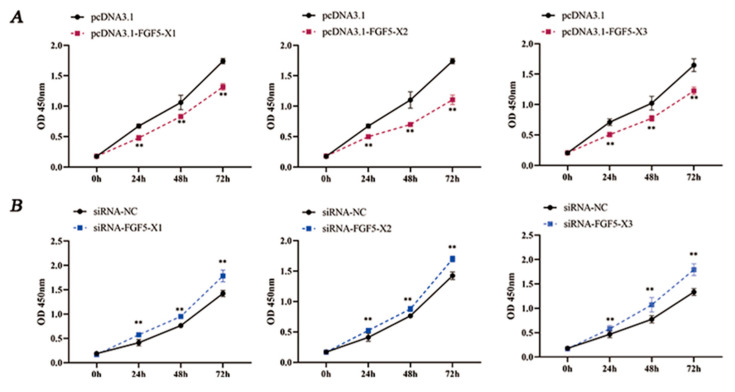
Cell proliferation after the overexpression and knockdown of FGF5 alternative spliceosomes. (**A**) Overexpression of FGF5 alternative spliceosomes inhibits cell proliferation. (**B**) Knockdown FGF5 alternative spliceosomes enhance cell proliferation. ** *p* < 0.01.

The role of FGF5 alternative spliceosomes in regulating DPC proliferation was determined. According to the results, the FGF5 alternative spliceosomes reduced DPC viability (*p* < 0.01, Figure 4A), whereas the knockdown of these spliceosomes enhanced DPC proliferation (*p* < 0.01, Figure 4B).

## 4. Discussion

FGF/FGFR are key regulators of cell proliferation, apoptosis, and differentiation. They are systematically involved in the pathophysiology of many human diseases, such as cancer and inherited metabolic diseases [33,34]. The long hair phenotype caused by *FGF5* gene mutation has been observed in cats, dogs, and donkeys [35,36,37]. With the development of research, growing evidence has shown that *FGF5* is a key signal molecule regulating the hair growth cycle. When the *FGF5* gene is damaged, it can prolong the growth period of hair follicles, thereby increasing the hair length. In cashmere goats, *FGF5* expression peaked at the resting stage of the hair follicle growth cycle, while *FGF5* peaked at the growth stage, indicating that *FGF5* gene plays a crucial regulatory role in the growth cycle [38]. *FGF5*, as a hair growth regulator, inhibits hair growth during the anagen phase. The FGF-5S protein significantly inhibited *FGF5* activity in macrophages and promoted hair growth on rat skin [39]. A missense mutation in *FGF5* caused large differences in hair growth between breeds of dogs [40] and rabbits [41]. The effect of the *FGF5* gene on hair growth has been extensively studied in many species. The *FGF5* gene affects hair length mainly by regulating the growth period of the hair follicles, but the effect of the *FGF5* gene on hair length is not limited to hair length. It also has an effect on the hair density of Rex rabbits. Three alternative spliceosomes of *FGF5* inhibited hair follicle growth in Angora rabbits [42]. Moreover, alternative spliceosomes have been identified in adenovirus studies [43], and they can serve as effective targets for cancer therapy [44], which provides a basis for the advancement of clinical trials in cancer research. Thus, we can conclude that the *FGF5* gene plays a regulatory role in hair growth. In our study, rabbit FGF5 alternative spliceosome coding sequences were cloned. The present study indicates that the spliceosome proteins are unstable and hydrophilic, as revealed by bioinformatics analysis. FGF5-X1, FGF5-X2, and FGF5-X3 encoded 267, 133, and 120 amino acids, respectively. According to the tertiary structure prediction diagram, the tertiary structure of the FGF5 alternative spliceosomes was quite different. Meanwhile, the spliceosomes contained no transmembrane domain and were not transmembrane transport proteins. Although predictions indicated that the FGF5 alternative spliceosomes contain putative phosphorylation sites, the role of phosphorylation in these spliceosomes needs to be explored further.

According to recent studies, alternative spliceosomes are involved in regulating body function. Different WNT4 alternative spliceosomes were constructed for research, and it was found that the alternative spliceosome product of WNT4-α played a key role in the WNT signaling pathway [45]. Furthermore, selective 5′ and 3′ are a subgroup of the ubiquitous selective splice-exon hopping form [46], and intradermally injected cholesterol-modified FGF5- or FGF18-targeting siRNAs prevented hair loss [47]. Different PRDM16 transcripts play different roles in leukemia hematopoiesis and the inflammatory reaction [48]. The variable spliceosome of follicle mRNA affects luteinizing hormone (LH) secretion in ewes with seasonal variations [49]. Tibetan sheep live in a high-altitude environment. Transcription factors related to LH and the follicle-stimulating hormone (FSH) significantly induced the differential expression of genes. The number of alternative spliceosomes increased, thus inhibiting follicle development [50]. From previous studies, it can be seen that the study of alternative spliceosomes is multifaceted and can affect the physiological function of individuals.

The evolutionary and structural biological features of *FGF5* may be related to its function in HF development [51]. Moreover, *FGF5* inhibitors are crucial as new drug candidates for human hair loss disorders. Verifying that FGF5 alternative spliceosomes influence HF growth, these spliceosomes played a regulatory role in the expression of genes such as *FGF2*, *LEF1*, and *CCND1*. Among the proteins, the cell cycle protein *CCND1*, a vital cell cycle regulator, promotes cell proliferation [52,53,54]. In the mouse population, *CCND1* knockout revealed that most mouse tissues developed normally, such as the kidneys, salivary glands, and seminal vesicles, which typically have readily detectable *CCND1* transcription levels. The hepatocytes of these mice still exhibited a proliferative response to mitotic stimulation, and cyclin E expression increased [55]. *CCND1* may mediate the proliferation of bulge stem cells into the more rapidly proliferating ORS cells [54]. Lymphoid-enhancer-binding factor 1 (*LEF1*) is a key nuclear transcription factor in the Wnt/β-catenin signaling pathway. In a series of epithelial tissues such as hair follicles and mammary glands, *LEF1* promotes normal development by regulating the interaction between epithelial cells and mesenchymal cells. During the growth period, *LEF1* expression in the outer root sheath (ORS) was slightly decreased along the hair bulb, mainly in the nucleus. *LEF1* expression in hair follicles decreased gradually and changed from a nuclear to a cytoplasmic expression [56]. During quiescence, *LEF1* appeared in the outermost layer of the bulge region, and its expression was also markedly enhanced in the inner root sheath below the bulge region, similar to the localization of stem cell markers. *BCL2* is an apoptosis-inhibiting gene and is considered an influential factor in tumor cell production and proliferation, and it may play a role in the HF growth cycle and morphogenesis [57,58,59,60]. Its expression in the follicular epithelium is dependent on the cycle. It is expressed in the epithelium, bulb, basal layer of the outer root sheath, and bulge in the most active part of circulating follicle during growth, but it is absent in the epithelium of quiescent follicles at rest, including the bulge [61]. Morphological and Western blot analyses revealed residual *BCL2* in the resting phase, mainly because of the presence of *BCL2* in the follicular papilla, the interstitial part of the follicles.

Fibroblast growth factor 2 (*FGF2*) has been reported to play various roles, such as affecting anxiety psychology [62], regulating diabetes and related complications [63,64], inducing tumor growth [65,66], and participating in the regulation of cell proliferation [67]. *FGF2* is a potent *FGF* of the epithelial mitogen and is involved in the signaling that is closely related to breast cancer development. This gene has been reported to play multiple roles, such as in influencing anxiety, regulating diabetes and related complications, inducing tumor growth, and participating in the regulation of cell proliferation [68]. According to some studies, the FGF2-FGFR signaling system affects breast development [69]. The FGF2-mediated signaling cascade plays a key role in tissue development and homeostasis, which can prevent stress, regulate mammary duct growth, and promote mammary epithelial cell proliferation and renewal [70]. FGF2- and FGF9-elicited FGF signaling was noted in a mouse population [71]. It could activate the ERK1/ERK2 pathway and promote cell proliferation and migration in the mouse mammary gland, thereby maintaining branches of the mammary epithelium [72]. It also participated in collagen recombination of fibroblasts. Sumbal et al. designed a primary mammary epithelial organoid to mimic the related processes of pregnancy and lactation. They found that *FGF2* initiates the mammary epithelium and enhances lactation in primary mammary organoids [71]. Transforming growth factor-β (*TGFβ*) can inhibit the growth of the hair follicle epithelium, but it can stimulate the proliferation of hair follicle dermal-derived cells. In the hair follicle growth cycle, the *TGFβ* receptor is strictly confined to the hair follicle epithelium on which the growth cycle of hair follicles was dependent [73].

## 5. Conclusions

In this study, we used bioinformatics methods to predict and analyze the physicochemical properties of alternative spliceosomes of the rabbit *FGF5* (*FGF5-X1, FGF5-X2,* and *FGF5-X3*). We found that all three alternative spliceosomes are unstable proteins, encoding 268, 134, and 121 amino acids, respectively, and have multiple phosphorylation sites. FGF5 alternative spliceosomes were successfully cloned, and their coding sequence lengths were 804, 402, and 363 bp, respectively. In the present experiment, overexpression or knockdown of the FGF5 alternative spliceosomes affected BCL2, CCND1, and FGF2 expression, indicating that the spliceosomes might regulate HF growth and development. In short, the FGF5 alternative spliceosomes can play a role in the growth of rabbit hair follicles. This study provides an understanding of the molecular mechanism underlying the role of FGF5 alternative spliceosomes in HF growth and development, and provides a theoretical reference for wool production improvement and treatment of hair-related illness.

## Figures and Tables

**Table 1:** Construction of the siRNA sequence using the plasmid vector.

Name	Primers (5′→3′)	Version
FGF5-X1	F: gggagacccaagctggctagcATGAGCTTGTCCTTCCTCCTCC	XM_008267686.3
	R: ggtttaaacgggccctctagaTCATCCAAAGCGAAACTTGAGTC	
FGF5-X2	F: gggagacccaagctggctagcATGAGCTTGTCCTTCCTCCTCC	XM_051819654.1
	R: ggtttaaacgggccctctagaTCATCCTCCCTTAGTGCTGGTT	
FGF5-X3	F: gggagacccaagctggctagcATGAGCTTGTCCTTCCTCCTCC	XM_008267687.3
	R: ggtttaaacgggccctctagaCTAAACTTGGCTTAACATATTGGCTT	

**Table 2:** Primer sequences.

Genes	Primer Sequence (5′→3′)
*FGF5-X1*	F: TTGGAAATATTTGCTGTGTCTCAGG
	R: TCGCTAAAAATTTGTTGCTGAAAAC
*FGF5-X2*	F: CGTCCCTCCTCCCTTACAGA
	R: GTCATCCTCCCTTAGTGCTGGG
*FGF5-X3*	F: GGTAGCACCTCGTCTTCCTC
	R: TCGTGGGAGCCATTGACTTT
*GAPDH*	F: CACCAGGGGCTGCTTTTAACTCT
	R: CTTCCCGTTCTCAGCCTTGACC
*LEF1*	F: GATCCTAGGCAGAAGGTGGC
	R: GCAGCTGTCATTCTTGGACC
*CCND1*	F: ACACGGACGTGGATTGTCTC
	R: GGTCCAGTTCATCCTCCGAC
*FGF2*	F: GTGTGTGCAAACCGTTACCTT
	R: TCGTTTCAGTGCCACATACCAG
*BCL2*	F: AACATCGCCCTGTGGATGAC
	R: GGCCGTACAGTTCCACGAA
*TGFβ*	F: CAGGTCCTGCGGAAGTCAA
	R: CTGGAACGGGCTCAACATCTA

**Table 3:** Analysis of FGF5 alternative spliceosome-encoded proteins.

Alternative Spliceosome	Amino Acid Quantity	Theoretical Isoelectric Point	Total Atom	Amino Acid Sequence Length	Fat Solubility Index
FGF5-X1	267	10.66	4138	267	64.72
FGF5-X2	133	10.01	1920	133	64.74
FGF5-X3	120	9.85	1720	120	69.25

## Data Availability

Data are contained within the article.
